# Rifampicin-Loaded Alginate-Gelatin Fibers Incorporated within Transdermal Films as a *Fiber-in-Film* System for Wound Healing Applications

**DOI:** 10.3390/membranes11010007

**Published:** 2020-12-23

**Authors:** Ameya Sharma, Vivek Puri, Pradeep Kumar, Inderbir Singh

**Affiliations:** 1 Chitkara College of Pharmacy, Chitkara University, Punjab 140401, India; ameya.sharma@chitkara.edu.in (A.S.); vivek.puri@chitkara.edu.in (V.P.); 2 Chitkara University School of Pharmacy, Chitkara University, Himachal Pradesh 174103, India; 3 Department of Pharmacy and Pharmacology, Faculty of Health Sciences, School of Therapeutic, Sciences, University of the Witwatersrand, Johannesburg, 7 York Road, Parktown 2193, South Africa; pradeep.kumar@wits.ac.za

**Keywords:** biopolymers, wound dressings, transdermal films, fibers, alginate, gelatin, rifampicin

## Abstract

The various biological and molecular cascades including different stages or phases such as inflammation, tissue proliferation, and remodeling phases, which significantly define the wound healing process. The natural matrix system is suggested to increase and sustain these cascades. Biocompatible biopolymers, sodium alginate and gelatin, and a drug (Rifampicin) were used for the preparation of fibers into a physical crosslinking solution using extrusion-gelation. The formed fibers were then loaded in transdermal films for wound healing applications. Rifampicin, an antibiotic, antibacterial agent was incorporated into fibers and afterwards the fibers were loaded into transdermal films. Initially, rifampicin fibers were developed using biopolymers including alginate and gelatin, and were further loaded into polymeric matrix which led to the formation of transdermal films. The transdermal films were coded as TF1, TF2, TF3 and TF4.The characterization technique, FTIR, was used to describe molecular transitions within fibers, transdermal films, and was further corroborated using SEM and XRD. In mechanical properties, the parameters, such as tensile strength and elongation-at-break (extensibility), were found to be ranged between 2.32 ± 0.45 N/mm^2^ to 14.32 ± 0.98 N/mm^2^ and 15.2% ± 0.98% to 30.54% ± 1.08%. The morphological analysis firmed the development of fibers and fiber-loaded transdermal films. Additionally, physical evaluation such as water uptake study, water transmission rate, swelling index, moisture content, and moisture uptake study were executed to describe comparative interpretation of the formulations developed. In vivo studies were executed using a full thickness cutaneous wound healing model, the transdermal films developed showed higher degree of contraction, i.e., 98.85% ± 4.04% as compared to marketed formulation (Povidone). The fiber-in-film is a promising delivery system for loading therapeutic agents for effective wound care management.

## 1. Introduction

Within the epithelial structure of the skin, a wound is a discontinuity, a disruption of the structure and function of underlying skin tissue. Different reasons can lead to a wound. The key causes of cuts and bites are cutting, abrasion, surgery, accidents, and burns [1]. Wound healing is a multifaceted physiological mechanism that restores the integrity of the skin, due to synchronized interactions between different biological structures [2]. It requires precisely organized steps associated with the presence of different types of cells at the wound site during different phases of healing process [3]. Interactions and timings between the patho-physiological processes in the wound healing process vary with acute and chronic wounds, whereas the primary stages retain the same [4].

The time-dependent stages involved in acute tissue healing are caused by tissue damages, these phases are haemostasis, the inflammation phase, the proliferation phase, and lastly, the tissue remodeling phase. All phases in the wound healing processes are four-fold dependent processes [5]. Wounds that fail to heal in a timely manner (4–8 weeks) are known as chronic wounds. Diabetes mellitus, various cardiovascular disorders, lack of proper nutrition, obesity, perpetuated bed rest, bacterial infections, etc., are the different factors that lead to chronic wounds [6]. Chronic wounds, due to the opening of the wounds, typically lose their protective shield and, by extending the inflammatory step of wound healing, microorganism colonies formation further hinders the healing process [7].

In United States, about 2% of the population accounts for 5.8 million people affected by wounds, and the cost of wound care is US$ 20 billion per year [8]. The healing process can be significantly inflated utilizing efficacious wound dressings which can be a combination of biopolymers and synthetic agents [9]. For direct skin touch, ideal wound dressings are designed to decrease the risk of infection, avoid debridement of wounds, and regulate unrestrained bleeding, in order to speed up the wound healing process [10]. Non-biocompatibility, causing more traumas on removal, impermeability to oxygen and moisture, requiring regular adjustments, costs, inability to avoid bacterial invasion, and excessive bleeding are the issues corresponding with wound dressings which are currently being used [11]. The modification and synthesis of biocompatible materials will create novel wound dressing applications [12].

In wound healing, biomaterials play a fundamental role [13]. For drug delivery, applications as the fibrous structures, including fibers and nanofibers, are considered acceptable carriers, hence fiber or nanofiber-loaded transdermal films could act as novel drug carrier in drug delivery [14]. Due to its soft and versatile properties, fiber and nanofiber-loaded transdermal films attributes as significant substitute for wound healing dressings [15]. Wound exudates are also absorbed easily by the fiber-loaded transdermal films. Various biopolymers, such as sodium alginate, gelatin, chitosan, collagen, and silk fibroins, are used for the development of suitable carrier system in wound healing applications and drug delivery [16,17].

In the present research, biopolymeric fibers of alginate and gelatin were loaded in gelatin films for developing a fiber-in-film system. This novel carrier system was fabricated initially with the development of fibers and then the loading of fibers into polymeric gel which led to the formation of transdermal films. Alginate, a biopolymer; has been preferred due to its high biocompatibility, reduced toxicity and cost, as well as moderate gelation property by incorporation of cationic divalent (Ca^2+^). It is an anionic polymer (natural origin), usually extracted from brown seaweed, and has been reported to be an extensively utilized polymer for biomedical applications and tissue engineering applications [18]. Distinct cross-linking preparation methods can be employed to develop hydrogel, and their resemblance to extracellular matrices (ECM) of living tissues enables wide-ranging applications in wound healing, bioactive agent delivery, such as small chemical drugs and proteins, and cell transplantation [19]. A physiologically moist microenvironment is preserved by alginate wound dressings, bacterial contamination at the wound site is reduced and wound healing encouraged [20]. Gelatin is a biopolymer and has been recognized as an effective wound healing agent as it allows rapid migration of healed cells and provides antibacterial properties [21]. In addition, because of its flexible biological function that involves the activation of the healing process within the regenerative and inflammatory phase, and its capacity to facilitate the healing process within the regenerative and inflammatory phase; it is non-toxic and plays an imperative role in the wound healing process [22].

A transdermal film or adhesive film, or a skin film, is used to administer a managed dosage of a medicinal substance through the skin over time. A skin film uses a special membrane to regulate the rate of the movement of the drug (stored in a reservoir inside the film) through the skin and into the bloodstream [23]. Transdermal films are a versatile formulation of different sizes comprising of one or more active substances and could be a suitable carrier for delivery. Transdermal films are smeared onto unbroken skin and even on wounds to provide an effective delivery of active substance to the systemic circulation through skin permeation [24]. Rifampicin, is an antibiotic agent, and has excellent antibacterial properties against Gram-positive and Gram-negative bacteria [25]. It is an antibiotic that is liposoluble or lipophilic in nature, thereby its entry is feasible in most tissues. The antibacterial mechanism of action relies on the inhibition of bacterial RNA polymerase based on DNA [26].

In addition, rifampicin is the only potent antibiotic against *Staphylococcus aureus*; rifampicin is an ideal antibiotic to be encapsulated into fibers and subsequently to films for its anti-bacterial properties and wound healing applications due to these excellent properties. Additionally, in physicochemical, physicomechanical, and morphological analysis; the developed fibrous systems were tested in vivo for their therapeutic potential in a full-thickness wound model. Within the alginate matrix, the fibers loaded transdermal films displayed an intricate molecular interaction profile, provided an irregular drug release mechanism, and improved wound healing compared to marketed formulation (Povidone).

## 2. Materials and Methods

### 2.1. Solvents and Reagents

The reagents used were sodium alginate, gelatin, calcium chloride, and rifampicin. Rifampicin was received as a gift sample from Banson Pharmaceuticals, Punjab, India. Sodium alginate, gelatin, and calcium chloride were purchased Sigma Aldrich, Bangalore, India. All solvents, reagents and chemicals (analytically graded) were used as received in the present research.

### 2.2. Experimental

#### Preparation of Rifampicin Fiber-Loaded Transdermal Films

**Figure 1:**
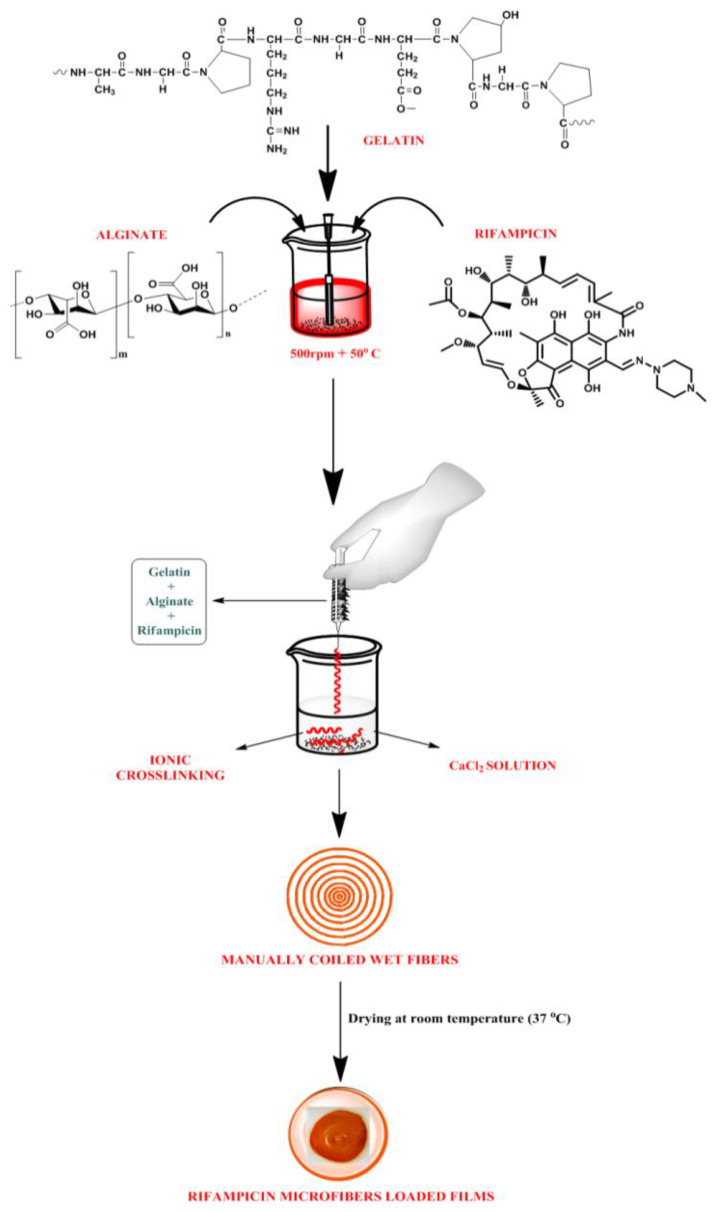
Fabrication method of rifampicin fiber-loaded transdermal films.

For the development of Rifampicin-loaded alginate-gelatin fibers, an ionotropic gelation method was used. Briefly, in weight ratios, sodium alginate: gelatin (1:1) solutions were prepared. The composition of fibers is depicted in Table 1, an aqueous biopolymeric solution was prepared by solublizing alginate and gelatin with the help of stirring for 30 min at 500 rpm. For complete solubilization of gelatin, the biopolymeric dispersions were heated to 50 °C. First, rifampicin (50 mg) was dissolved in 5 mL of acetone and then slowly incorporated to the polymeric solution. The mixture of rifampicin/alginate/gelatin was then extruded into a beaker containing 1wt% CaCl_2_ and fibers developed by ionic crosslinking (using a 22 gauge needle) [27]. The developed rifampicin fibers were then coiled manually and poured into petri-plate containing alginate solution (2%) and Polyvinyl Alcohol (PVA) (0.25%), as illustrated in Figure 1. The coiled fiber-loaded polymeric solution was dried at room temperature for 24 h and transdermal films were kept in a desiccator. Various formulation batches (TF1, TF2, TF3 and TF4) were prepared as depicted in Table 2.

## 3. Physicochemical Evaluation of Fiber-Loaded Transdermal Films

### 3.1. Film Thickness and Weight Variation

The digitalized micrometer (Mitutoyo, Kawasaki, Japan) was employed to measure the thickness of sample films. Average thickness and standard deviation of three readings were noted. For weight variation test, the sample films were weighed individually and results were determined using average ± SD.

### 3.2. pH Measurement

The pH of the prepared film-forming solutions was determined with the help of pH meter. The pH meter was firstly calibrated before use with buffered solutions at varied pH (4, 7 and 10).

### 3.3. Determination of Drug Content

The drug distribution uniformity was assessed by measuring drug content (%) at varied regions of the film by spectrophotometric method. The sample films with predetermined area were initially dispersed in 100 mL solution, the solution was comprised of 50 mL pH 7.4 phosphate buffer and 50 mL ethanol. The solution was agitated on orbital shaker for 24 h. Afterwards, the solution was filtered, diluted and measured at 337 nm employing UV-Visible spectrophotometer (2202, Systronics, India). This study was executed thrice, the average value and standard deviation (average ± SD) were recorded.

### 3.4. Moisture Content

The sample films were initially weighed and referred as (Wi) and were put into desiccator which was consisting of activated silica gel at 25 °C for 24 h. The films were weighed individually and repeatedly until the constant weight (Wd) was observed and the moisture content was determined according to the equation given below:

(1) Moisturecontent % = [(Wi−Wd)/Wd] ×100

### 3.5. Moisture Uptake Study

The sample films were initially weighed and referred as (Wi) put into a desiccator which was containing activated silica gel at 25 °C for 24 h. The films were transferred to another desiccator which contained a saturated NaCl solution at relative humidity (75% ± 25 °C). The films were weighed individually and repeatedly until the constant weight (Wm) was observed and the moisture uptake capacity was determined according to the equation given below:

(2) Moistureuptakecapacity % = [(Wm−Wi)/Wi] ×100

### 3.6. Folding Endurance

The folding endurance was deliberated by folding the film repeatedly at the same place until it became disrupted. The films could be easily folded a number of times at same place, without breaking; this was the folding endurance value.

### 3.7. Mechanical Properties

The sample films were analyzed, employing a texture analyzer (TA XT plus, Stable Microsystem, Godalming, UK) for its mechanical properties, and provided with 5 kg of loaded cell. Film of size 1cm^2^ was cut and clutched between both the clamps and placed at a distance of 10 mm and forcibly separated at 50 mm/min rate. The two parameters, i.e., tensile strength and extensibility were evaluated thrice of each batch of sample films. The parameters were manifested in N/mm^2^ and percentage individually.

### 3.8. Swelling Index

The dried sample films were pre weighed and dispersed in 250 mL phosphate buffer with specifications (pH 7.4 at 25 °C). The swelling ratio (Q) of the sample films was determined, applying the below equation:

(3) Q=Ws/Wd

where Ws depicts the weight of swollen films at varied time intervals and Wd is weight of dried films.

### 3.9. Water Vapour Transmission Rate (WVTR)

The sample film was mounted on the top of a polytop glass (144 mm^2^) containing a phosphate buffer of 10 mL (pH 7.4). The sample films were pre-weighed and put in an oven for 24 h at 35 °C. Using the following equation, WVTR was determined.

(4) $$\mathrm{WVTR}=\mathrm{Wi}-\frac{\mathrm{Wt}}{A}\times 106\;g/m^{2}{\mathrm{day}}^{-1}$$

If WVTR is expressed in g/m^2^/h, A = polytop opening area (mm^2^), Wi and Wt = polytop weight before and after being put in the oven, respectively.

## 4. Characterization of Fiber Loaded Transdermal Films

### 4.1. Morphological Analysis

Morphological examination of selected formulations was executed by a scanning electron microscope (S 4300 SE/N, Hitachi, CA, USA) with an accelerating voltage of 15kV. All the sample films were staged on a metallic stub and adhered with double side tape, and then further coated with a golden layer.

### 4.2. XRD

X-ray diffraction pattern of selected formulations were determined employing X-ray diffractometer (P analytical X’Pert Pro MRD, Malvern Panalytical Ltd., Malvern, UK) under specification such as scanning between 2 theta of 0–60° and counting time is 0.001 s.

### 4.3. FTIR

A FTIR spectrophotometer (IFS66/S, Alpha Bruker, Ettlingen, Germany) was used to determine FTIR spectra of the selected formulation. FTIR spectra of prepared film formulations were mixed with KBr and then compressed. The pellets were analyzed in the spectral range of 4000–400 cm^−1^.

### 4.4. In Vitro Release Studies

The sample films comprising 50 mg equivalent wt of Rifampicin and were mounted to a glass slide and affixed to a mesh screen (stainless steel). This assembly was securely placed at the bottom of dissolution test apparatus (Paddle type-Lab India DS 8000, Mumbai, India). Buffer solution (Phosphate buffer 7.4) and ethanol, in the ratio of 1:1, was used as a dissolution medium. The test conditions with standard specifications w.r.t temp and speed (32 °C and 50 rpm) were provided to the medium. The samples (5 mL) were taken out at a framed mean time, further the samples were analyzed at 337 nm utilizing a UV/Visible spectrophotometer (2202, Systronics, Mumbai, India). In vitro drug release data was fitted into various kinetic models, such as Zero order, First order, Higuchi, Hixon–Crowell, and Korsmeyer–Peppas model, for understanding the mechanism of drug release from the formulation.

(5) $$Q=Q_{0}\;+{\;k}_{0}t\;(\mathrm{Zero}\;\mathrm{order})$$

(6) Q= kHt12 (Higuchi model)

(7) $$\ln Q=\ln Q_{0}\;+{\;k}_{1}t\;(\mathrm{First}\;\mathrm{order}).$$

(8) Q013−QR13 =kst (Hixson-Crowell model)

(9) $$Q/Q_{T}=k_{\mathrm{kp}}t^{n}\;(\mathrm{Korsmeyer}-\mathrm{Peppas}\;\mathrm{model})$$

where Q is amount of drug release at time t, Q_0_ is the initial amount of drug, Q_R_ is the amount of drug remaining at time t, and Q_T_ is the total amount of drug release, k_0_, k_1_, k_H_, k_s_ and k_kp_ are the kinetic constants for zero order, first order, Higuchi, Hixson–Crowell and Korsmeyer–Peppas models, respectively, and n is the release exponent.

### 4.5. In Vitro Skin Permeation

In vitro skin permeation studies were performed, plying Franz diffusion cell apparatus which contained possessing receptor compartment and having capacity of 20 mL. From the dorsal surface, the full thickness rat skin was excised and deployed for permeation studies. The rat skin was placed over the receptor compartment. The sample (5 mL) was taken out at framed intervals and were investigated at 337 nm utilizing UV Visible spectrophotometer (2202, Systronics, Mumbai, India).

### 4.6. Antimicrobial Studies

In order to determine the antimicrobial activity of formulated transdermal films, the agar disc method was used. In this method, *Staphylococcus aureus* (gram positive bacterium) and *Escherichia coli* (gram-negative bacterium) which are predominantly found in the wound bed were used for the study. Agar solution was formulated using the procedure set out in Hi Media. In brief, 28 g of powder was introduced to 1000 mL of purified water. The solution was heated to boil so that the medium was dissolved completely, and further sterilized for 15 min by autoclaving at 121 °C. Cooling of up to 40–50 °C was permitted and the agar plates were prepared by pouring 20 mL of liquid agar media. The bacterial culture suspension of *E. coli* was inoculated into agar plates to prevail static growth. Petri plates were conceded to get solidified and 6 mm pit was produced plying a sterilized borer. The drug-containing control group (rifampicin) and transdermal film (TF4) were put in an agar plate pit and incubated at 37 °C for 24 h, and the zone of inhibition was deliberated.

### 4.7. In Vivo Animal Studies

In vivo animal study was carried out in compliance with the protocol approved by the Chitkara College of Pharmacy’s Animal Ethics Committee, Chitkara University, India (CPCSEA registration number: 1181/PO/REBI/S/08/CPCSEA). Ketamine (80 mg/kg) was used to anaesthetize healthy male rats weighing 220–250 g. The specific area of the skin was shaved with the help of an epilator for approximately 200 mm^2^ of wound production. There were 20 animals divided into five groups: control group, rifampicin fibers, transdermal film (TF2), transdermal film (TF4), and marketed formulation (Povidone). The transdermal film (TF2), (TF4), and rifampicin fibers were applied to the wound for proper covering wound beds. The marketed formulation (povidone) was also applied as a commercial product and the wound lesion size was captured and observed at a close distance on day 0, 2, 4, 6, 8, 10, 12, and 14. The wound dressings were changed regularly on every 4th day. With the help of calibrated vernier caliper, wound contraction area was measured. The following formula was used to measure the percent of wound contraction:

(10) % Wound contraction=Initial wound area−specific day wound area×100/Initial wound area

## 5. Results and Discussion

### 5.1. Physicochemical Evaluation of Transdermal Films

The results of various physicochemical characterization studies of transdermal films are incorporated in Table 3. The transdermal film thickness was reported to range from 0.038 ± 0.006 mm to 0.043 ± 0.007 mm. The weight of the various film batches ranged from 0.043 ± 0.007 g to 0.472 ± 0.08 g, which reveals that TF4 comprised of fibers weights were relatively higher. It was found that the drug content of formulated transdermal films was 96.04 ± 0.56% to 98.92 ± 0.88% in TF2 and TF4. The pH of the formulations ranged between 6–8, which is considered as an ideal pH range for transdermal drug delivery.

Moisture content is a parameter which is used as an indicator to determine amount of water that a film contains. The drug content was found to be ranged between 96.04 ± 0.56% to 98.92 ± 0.88% proclaiming consistency of manufacturing process.

The moisture content was found to be ranged between 12.21 ± 0.79% to 17.12 ± 0.98%. Regarding the protection of formulations from microbial contamination or exposure and also to decrease bulkiness; the percent of moisture uptake of the transdermal films should be low. The percentage values of moisture absorption ranged from 12.08 ± 0.82% to 14.68 ± 0.82%.

The folding endurance tests evaluate the film’s ability to endure breakage. The folding resistance ranged from 232 ± 14 to 298 ± 10 folds for the transdermal films. Compared to other transdermal films, formulations consisting of fibers displayed higher values.

#### 5.1.1. Mechanical Properties

Pertaining to various parameters such as tensile strength and percent elongation to break (extensibility), the mechanical properties of the transdermal films were evaluated. Pure alginate film (TF1) demonstrated 2.32 ± 0.45 N/mm^2^ tensile strength and 15.2 ± 0.98% extensibility. It was found that tensile strength and formulation extensibility were 2.54 ± 0.82 N/mm^2^ and 16.28 ± 0.52%, 14.18 ± 0.76 N/mm^2^ and 29.18 ± 1.03% in TF2 and TF3, respectively. The transdermal film of TF4 tensile strength and extensibility were found to be 14.32 ± 0.98 N/mm^2^ and 30.54 ± 1.08%, respectively as depicted in Table 4. The mechanical properties were further improved by the incorporation of fibers, this increase in mechanical properties may be attributable to the physically cross linked networks between the alginate and gelatin polymeric matrix and fibrous matrix interfacial affinity interaction. Dong et al. reported that alginate gelatin blended films showed higher value of tensile strength and % elongation at break, the results indicated that blending of the polymers were relatively efficacious in improving the mechanical properties [28].

#### 5.1.2. Swelling Ratio

The swelling ratio was ranged to be 1.92 ± 0.56% and 4.42 ± 9.68% for TF1 and TF4, respectively, as shown in Table 4. With the addition of fibers, the rise in the swelling ratio may be attributed to an increase in hydrophilic groups of films. In addition, physical entanglement of the polymeric chains of alginate and gelatin fibers contributes to the creation of a hydrogel network. Chiaoprakobkij and co-workers have developed wound dressing based on biopolymers, and reported that the biopolymeric network and entanglement of polymer matrix led to an increase in swelling index [29].

#### 5.1.3. Water Vapor Transmission Rate (WVTR)

The results of this parameter was ranged between 684 ± 1.86 g/m^2^ and 808 ± 1.06 g/m^2^, which may be attributed to the excessive gelling property of polymers and the addition of fibers contributing to the narrowing or closing of the pores and channels responsible for water vapor transmission. Furthermore, the addition of fibers leads to pores and channel closure, leading to a drastic reduction in transmission rate in transdermal film, as depicted in Table 4. Shi and his research team developed polymeric nanofibers loaded polymer based films for drug delivery applications. Researchers have suggested that nanofibers incorporation into polymeric matrix led to the narrowing of the channels as gelling property was exhibited in the matrix system [30].

### 5.2. Characterization of Transdermal Films

#### 5.2.1. Morphological Analysis

**Figure 2:**
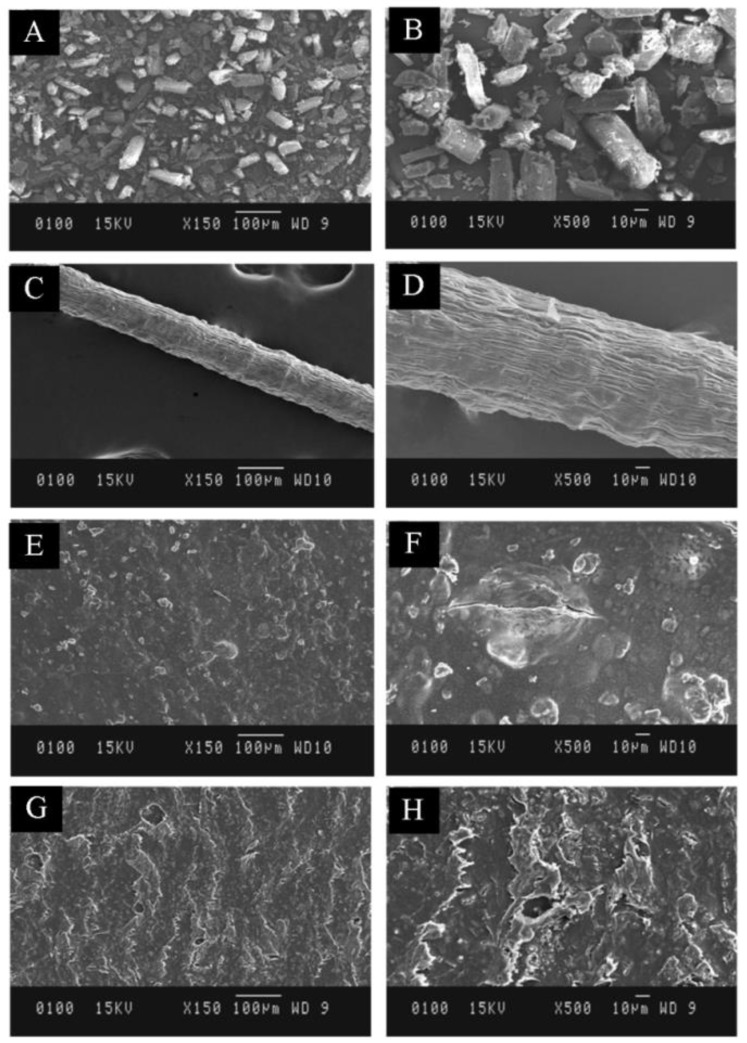
SEM images of different batches at magnification value (**A**) is 150× of drug; (**B**) is 500× of drug; (**C**) is 150× of fiber; (**D**) is 500× of fiber; (**E**) is 150× of transdermal film (TF2); (**F**) is 500× of transdermal film (TF2); (**G**) is 150× of transdermal film (TF4); (**H**) is 500× of transdermal film (TF4).

SEM representations of various batches of formulated transdermal films as shown in Figure 2, in the polymer matrix of formulated transdermal films. The rough surface topology with small rod shape affirms consistent disposition of drug used (rifampicin). The SEM images further indicated the presence of intercalated lamellar structures in TF2 and TF4 was observed as fibers were being trapped within the polymer matrix. The surface of fiber manifested smooth and uniform morphology that evidently indicates magnified miscibility and uniform homogeneity between both the polymers.

#### 5.2.2. XRD

**Figure 3:**
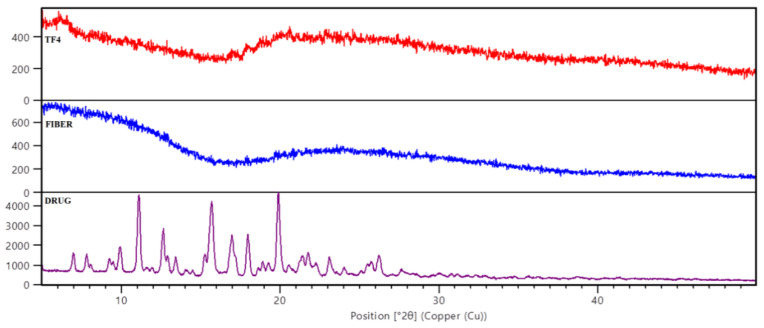
X-ray Diffraction pattern of drug, fibers, and transdermal films (TF4).

**Figure 4:**
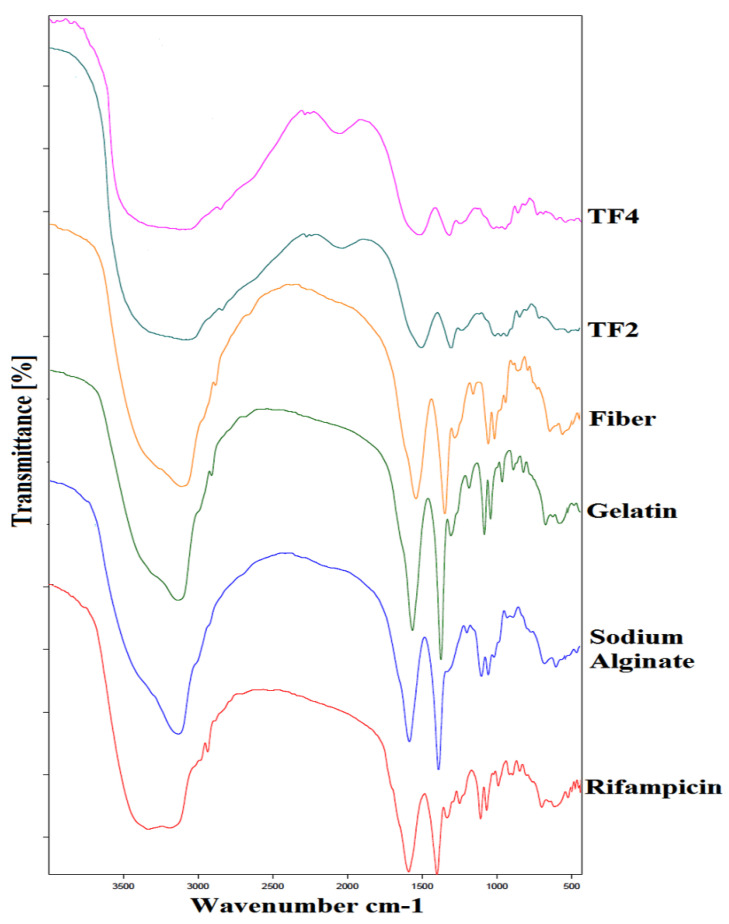
FTIR spectra of drug (rifampicin), sodium alginate, gelatin, fibers (rifampicin loaded), fiber-in-film TF2 batch, and fiber-in-film TF4 batch.

According to the results of the XRD pattern (Figure 3), rifampicin has a crystalline nature and displays substantial peaks at 2θ = 11, 12.8, 15.9, 16.2, 17.1, 18, and 20, respectively, as shown in Figure 4. The loss of crystalline peaks was observed in developed fiber and transdermal film (TF4). Intercalation and possible bonding between the polymers were indicated in the findings, hence it confirmed that the drug was successfully lodged between the matrix of polymer in fibers, as well as in the reservoir system of transdermal film. Gajendiran and his co-workers reported that rifampicin is crystalline natured and showed prominent peaks at 2θ whereas in the formulation developed, no such peaks were observed [31].

#### 5.2.3. FTIR

Fourier transform infrared (FTIR) attenuated total reflectance was used to obtain detailed information on the interaction seen between formulations. Figure 4 displays the FTIR spectrum of rifampicin, sodium alginate, gelatin, rifampicin loaded fibers, fiber-in-film TF2 batch and fiber-in-film TF4 batch. At 3313 cm^−1^ (OH Stretching), 1608 cm^−1^ (Carboxylic C=O), 1291 cm^−1^ (C–CH), 1088–1051 cm^−1^ (C–O Stretching), 1024 cm^−1^ (C–C), 949cm^−1^ (C–O), 880cm^−1^ (CH), and 812 cm^−1^ (Na–O), the IR spectrum of alginate display characteristic absorption bands [32]. At 3411 cm^−1^ (NH stretching), 1650 cm^−1^ (amide I, C=O), 1549 cm^−1^ (amide NH bending) and 1335 cm^−1^ (for C–N stretching), the significant gelatin peaks showed absorption bands. The FTIR spectra of drug rifampicin showed peak at 3479 cm^−1^ (NH stretching), 2893 cm^−1^ (C-H bonding), 1627 cm^−1^(C=O), 1478 cm^−1^(C=C), 1376 cm^−1^ (CH2, C=C), 1053 cm^−1^ (–CH, CO, C–H), 984 cm^−1^ (≡C–H, C–H) [33]. The FTIR spectrum corresponding to rifampicin loaded fibers and fibers-in-film system were identical to that of the polymers used for preparing the formulations. Since the characteristic peaks of rifampicin were missing in FTIR spectra of fibers and fiber-in-film, it could be deduced that rifampicin was well embedded in the matrix of fibers and reservoir of fiber-loaded transdermal films.

#### 5.2.4. In Vitro Drug Release

**Figure 5:**
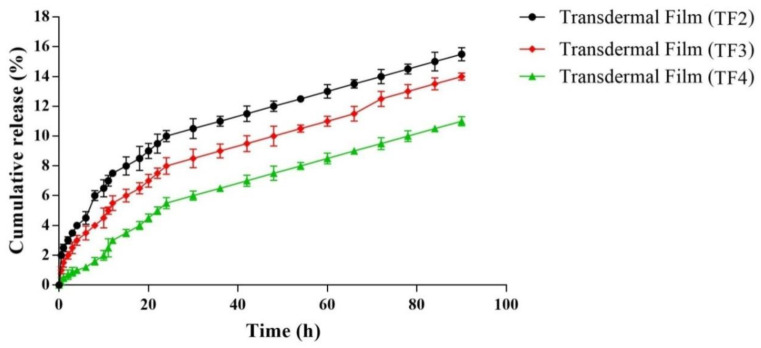
In vitro release of various batches of transdermal films.

The findings of the in vitro drug release analyses showed that transdermal films, loaded with fibers, have higher polymeric concentrations, as the polymer was used both as polymeric gel and for fabrication of fibers, hence the cumulative percent release of drugs decreases. As shown in Figure 5, the drug releases from TF2, TF3, and TF4 were found to be 16.22% ± 4.32%, 14.48% ± 3.28% and 10.24% ± 3.2% in 90 h, respectively. The formation of the polymer matrix by alginate could be a retardation factor of releasing the drug from formulated transdermal films. Adding fibers further retards the release of drugs from formulated transdermal films. This may be due to the interpenetration in the polymeric chains of fibers and polymer matrix of alginate. The interference of fibers with the relaxation of the polymeric chain (up to hydration) could also lead to a reduction in the release of drug from TF4. Several kinetic models were fitted with the drug release data obtained after an in vitro release analysis, i.e., first order, zero order, model Higuchi, Hixon–Crowell and Korsmeyer–Peppas. For the selected formulations, as shown in Table 5, the value of n was found to range from 0.54 to 0.75, suggesting an anomalous non-Fickian drug release mechanism from the formulated films. In addition, the process mediated by diffusion and erosion may be responsible for the release of the drug from transdermal film.

#### 5.2.5. In Vitro Permeation

**Figure 6:**
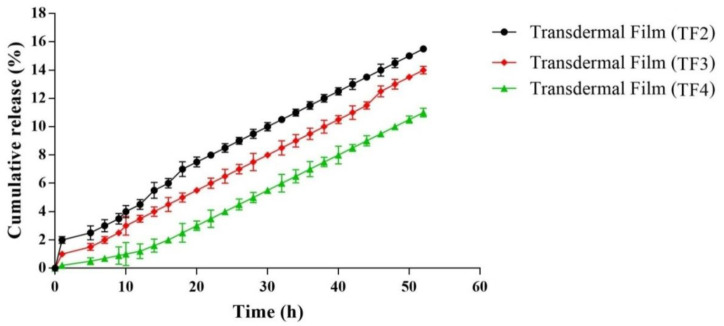
In vitro permeation of various batches of transdermal films.

The findings of the in vitro permeation studies of animal skin from diffusion membrane are similar to in vitro drug release studies. In TF2, a polymer matrix produced by alginate has less water affinity; and resulted in the decrease in thermodynamic activity of the drug in the diffusion membrane, further leading to the decreased drug release from the film. The addition of fibers further delayed the release of the drug from the formulation in TF3 and TF4, as depicted in Figure 6.

#### 5.2.6. Antimicrobial studies

**Figure 7:**
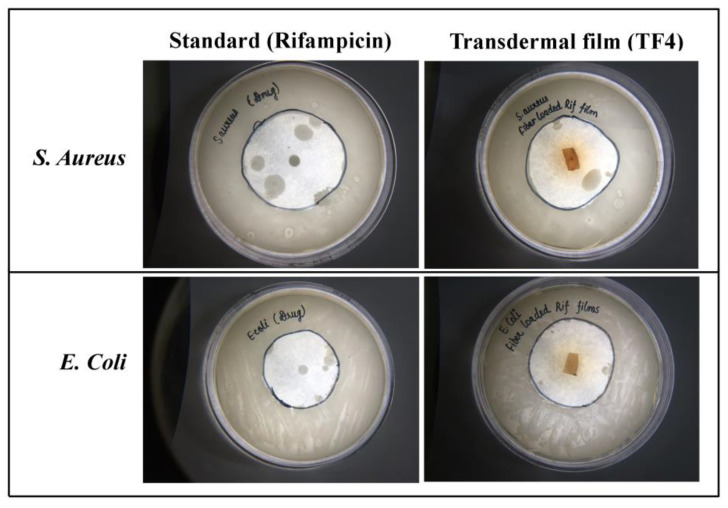
Antimicrobial activity of standard group (Rifampicin) and transdermal film (TF4) against *S. aureus* and *E. coli*.

The antimicrobial activity of rifampicin against gram +ve and gram –ve bacteria was evaluated using disc diffusion method. The zone of inhibition of transdermal film (TF4) was found to be 24 mm against *S. aureus*, and 23 mm against *E. coli*; in contrast; standard or control group (Rifampicin) resulted to have a zone of inhibition 25 mm against *S. aureus* and 21 mm against *E. coli.* The antimicrobial studies revealed that the drug was successfully released from the polymeric matrix of fibers and reservoir of fiber-loaded transdermal films, and showed antimicrobial activity as shown in Figure 7.

#### 5.2.7. In Vivo Studies

**Figure 8:**
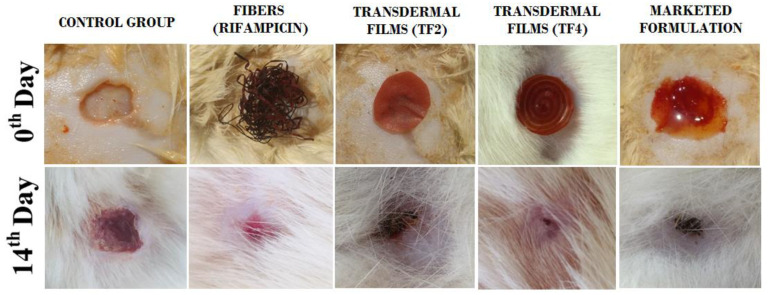
Wound healing process on the 0th and the 14th day of treatment with control group, fibers (Rifampicin), transdermal films (TF2), transdermal films (TF3) and marketed formulation (Povidone).

**Figure 9:**
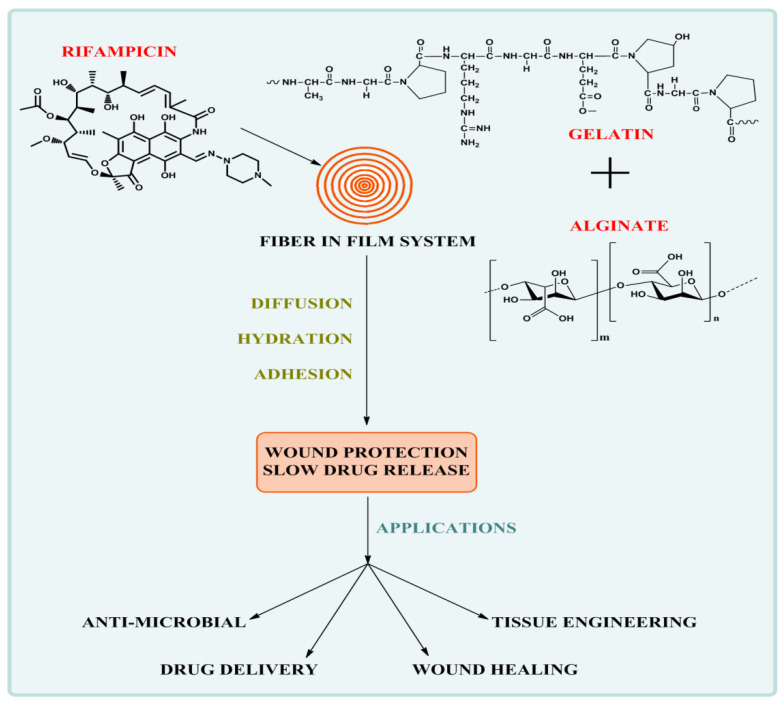
Scheme representing the actions and applications of fiber-in-film system.

An animal study was executed, in accordance with protocol authorized by Animal Ethics Committee, Chitkara College of Pharmacy, Chitkara University, Patiala, Punjab. It was observed that in the treatment group, the wound contraction developed faster as compared to the control group. Here, between the 10th and 14th days, absolute wound healing was observed. An indication of the wound healing property is the percentage degree of wound contraction. Transdermal film (TF4) showed 82.91 ± 2.78 degree of contraction, Transdermal film (TF2) showed degree of contraction 81.96 ± 2.73, Rifampicin fibers showed 80.92 ± 2.72 degree of contraction whereas the commercial formulation (Povidone^®^) showed 91.87 ± 3.72 degree of contraction. It may be due to proper intimation of wound dressing onto the wound bed, the drug was released from transdermal films sustainably. In the control group, the degree of contraction was found to be 58.83 ± 2.78 as given in Table 6. Hair growth was observed on the 10th day in the transdermal films group, fibers, and marketed formulation, but, in the control group, the hair growth was not observed till the 14th day.TF4 exhibited faster wound healing properties compared to the control and TF2 formulation batch. Regulated release of Rifampicin from the formulations may be due to the accelerated wound contraction from formulated transdermal films, as shown in Figure 8. The imbibition and diffusion of wound fluid will cause hydration and subsequent adhesion of the fiber-in-film system on to the wound. Furthermore, the transdermal system would be responsible for slow drug release for wound healing and tissue engineering applications. A schematic representation of the actions and applications of fiber-in-film system are depicted in Figure 9.

## 6. Remarks and Conclusions

A suitable analytical method of rifampicin was developed by UVVisible spectrophotometer and in a mixture of phosphate buffer (pH 7.4) and ethanol (50:50, *v/v*), rifampicin showed maximum absorption at a wavelength of 337 nm. The formulation developed was characterized by various techniques such as SEM, XRD, and FTIR, and resulted as absence of interaction between drug and polymeric matrix. The kinetic study was executed and fitted into various kinetic models where n value depicted anomalous behavior of all formulations developed. The formulation batches have maximum release at 90 h as controlled drug delivery system. In animal study, the degree of contraction was found to be higher in formulated transdermal films, as compared to marketed formulations. The formulated fiber-loaded transdermal films could be a suitable carrier system for drug delivery in biomedical applications and wound healing applications.

## Figures and Tables

**Table 1:** Composition of fibers.

Formulation	Alginate	Gelatin	Drug	CaCl_2_
Fibers	2%	2%	50 mg	1%

**Table 2:** Composition table of rifampicin fibers loaded transdermal films.

Formulation Code	Rifampicin in Polymeric Solution (50 mg)	Alginate (2% *w/w*)	PVA (0.25% *v/v*)	Rifampicin Loaded Fibers (Incorporated)
**TF1**	x	✓	✓	x
**TF2**	✓	✓	✓	x
**TF3**	x	✓	✓	✓
**TF4**	✓	✓	✓	✓

**Table 3:** Physicochemical evaluation of transdermal films.

Formulation Batches	Thickness (mm)	Weight Variation (g)	Drug Content (%)	Moisture Content (%)	Moisture Uptake (%)	Folding Endurance (Folds)
TF1	0.038 ± 0.006	0.382 ± 0.03	-	17.12 ± 0.98	14.68 ± 0.82	232 ± 14
TF2	0.039 ± 0.003	0.424 ± 0.05	96.04±0.56	15.14 ± 0.94	14.42 ± 0.72	268 ± 9
TF3	0.041 ± 0.007	0.468 ± 0.04	97.61±0.42	12.62 ± 0.92	12.42 ± 0.61	288 ± 12
TF4	0.043 ± 0.007	0.472 ± 0.08	98.92±0.88	12.21 ± 0.79	12.08 ± 0.82	298 ± 10

**Table 4:** Mechanical properties and swelling ratio of transdermal films.

Formulation Batch	Swelling Ratio (%)	Tensile Strength (N/mm^2^)	Extensibility (%)	WVTR (g/m^2^/h per Day)
TF1	1.92 ± 0.56	2.32 ± 0.45	15.2 ± 0.98	798 ± 1.28
TF2	1.82 ± 0.42	2.54 ± 0.82	16.28 ± 0.52	808 ± 1.06
TF3	4.40 ± 0.84	14.18 ± 0.76	29.18 ± 1.03	684 ± 1.86
TF4	4.42 ± 9.68	14.32 ± 0.98	30.54 ± 1.08	692 ± 2.23

**Table 5:** In vitro drug release (kinetic modeling) data of various transdermal film batches.

Batches	Zero Order		First Order		Higuchi Model		Hixson Crowell Model		KorsmeyerPeppas Model		
	r^2^	k_o_	r^2^	k_1_	r^2^	k_H_	r^2^	k_HC_	r^2^	k_kp_	n
**TF2**	0.863	0.143	0.962	−0.013	0.981	1.554	0.968	−0.016	0.989	0.391	0.414
**TF3**	0.918	0.137	0.981	−0.009	0.995	1.456	0.979	−0.012	0.996	0.167	0.503
**TF4**	0.951	0.121	0.960	−0.005	0.986	1.254	0.949	−0.008	0.985	0.400	0.760

**Table 6:** Degree of Contraction data of control, fibers, transdermal films and marketed formulation.

Groups	Day 2	Day 4	Day 6	Day 8	Day 10	Day 12	Day 14
Wound (control group)	17.34 ± 1.11	21.71 ± 2.87	33.82 ± 2.07	44.78 ± 1.88	58.83 ± 2.78	72.57 ± 5.82	87.74 ± 2.97
Fibers (Rifampicin)	24.32 ± 4.95	37.74 ± 3.96	57.92 ± 1.92	67.85 ± 3.05	80.92 ± 2.72	96.79 ± 6.43	98.78 ± 4.02
Transdermal film (TF2)	23.32 ± 4.91	35.42 ± 3.92	55.92 ± 1.97	67.81 ± 3.04	81.96 ± 2.73	95.71 ± 6.51	98.72 ± 4.01
Transdermal Film (TF4)	25.39 ± 4.97	38.77 ± 3.97	58.97 ± 1.96	68.88 ± 3.07	82.91 ± 2.78	97.78 ± 6.57	98.85 ± 4.04
Marketed formulation (Povidone)	33.68 ± 3.97	48.97 ± 4.04	58.09 ± 5.04	78.94 ± 4.07	91.87 ± 3.72	96.42 ± 2.93	97.12 ± 3.56

## Abbreviations

nm	Nano meter
N/mm^2^	Newton per square millimeter
Wi	Initial weight
Wd	Constant weight
TF	Transdermal films
PVA	Poly-vinyl alcohol
SD	Standard deviation
UV	Ultra violet
WVTR	Water vapor transmission rate
kV	Kilo volt
%	Percent
Ɵ	Theta
